# Empowering medical educators to teach planetary health: development and outcomes of a curriculum integration training

**DOI:** 10.1186/s12909-026-10170-5

**Published:** 2026-08-18

**Authors:** Hélène Furaha Hauch, Nadja Kutschke, Angela Schuster, Sabine Gehrke-Beck

**Affiliations:** Charité – Universitätsmedizin Berlin, Corporate Member of Freie Universität Berlin, Humboldt-Universität zu Berlin and Berlin Institute of Health, Institute of General Practice, Charitéplatz 1, Berlin, 10117 Germany

**Keywords:** Medical education, Climate change, Faculty training, Lecturer training, Planetary health, Curriculum, Change agency

## Abstract

**Background:**

Planetary Health (PH) content is increasingly recognized as essential for medical education. For longitudinal integration across the curriculum, medical educators can serve as change agents and multipliers. A targeted training that conveys both PH knowledge and pedagogical skills can empower medical educators to incorporate PH topics into their teaching.

**Methods:**

Following the curriculum development cycle by Kern, we developed a training for medical educators of Charité – Universitätsmedizin Berlin teaching in the medical curriculum. The learning objective was to enable medical educators to effectively integrate PH content into their courses.

The program was delivered in a blended-learning format: input on the interconnectedness between human and planetary health was provided online via a learning platform prior to the in-person unit (1 teaching unit [TU]). The face-to-face workshop (6 TU) gave input on Planetary Health as well as didactic input and facilitated discussion of personal experiences with climate change and its relevance to teaching, thereby enhancing participants’ change agency. Further, participants developed teaching materials for their own courses and tested them via a micro-teaching exercise. As follow-up the participants revised their teaching materials-based feedback on their micro teaching (1 TU). The impact of the workshop was evaluated according to Kirkpatrick’s framework.

**Results:**

Eighteen medical educators participated across three workshops, with 13 completing the follow-up questionnaire. Seventeen participants (94% response rate) completed the post-workshop evaluation. Participants reported satisfaction with the content, structure, and practical relevance of the course and rated their knowledge gain as moderate to high. Fifteen participants intended to integrate the developed teaching materials into their courses, and 14 planned to share their materials with colleagues. One year later, nine participants (50%) responded to a follow-up survey; five had integrated the materials into their teaching, and three had shared them with colleagues.

**Conclusion:**

A targeted training that actively prepares and supports the transfer of knowledge into teaching practice is well received by medical educators. PH content was integrated into courses by a subset of participants. Beyond the workshop, ongoing networking may further support sustainable PH content integration into the curriculum.

**Supplementary Information:**

The online version contains supplementary material available at 10.1186/s12909-026-10170-5.

## Introduction

### What is the problem?

Climate change, biodiversity loss, and air pollution are increasingly affecting human health and healthcare systems. Health professionals must therefore engage with these impacts as part of their professional responsibilities [1, 2]. Planetary Health (PH) is a framework that interconnects human health with the integrity of Earth’s natural systems [3], encompassing multiple dimensions of health. There is broad consensus across academia and civil society actors that PH content should be integrated into the curricula of health professions education [4, 5]. However, actual implementation within medical curricula remains limited and insufficient [6].

In Germany, specific learning objectives related to PH have been defined and incorporated into the National Competency-Based Learning Objectives Catalogue for Medicine (NKLM) [7]. In addition, various educational projects have been described and implemented to promote curricular integration [7–9]. Despite these efforts, a nationwide survey indicates that PH teaching is still frequently confined to elective courses, thus primarily reaching students with a strong intrinsic motivation for the topic [8, 10]. However, longitudinal integration into the core curriculum, is considered more effective and adequate to prepare physicians for the health impacts of climate change in clinical practice [11–13].

While barriers to curricular integration include increasingly dense medical curricula, which limit the incorporation of additional teaching content [11], medical educators have been identified as key stakeholders as their subject-matter expertise, attitudes, and time availability significantly influence the success of PH implementation into the curriculum [12, 14].

### Importance of change agency for PH in curricular transformation

Sustainable curricular change in medical education depends not only on structural frameworks but also on individuals who actively drive implementation. Change agency, as defined by Fullan (2007) [15] and Priestley et al. (2015) [16], refers to the capacity to initiate and sustain change within complex systems by translating goals into practice. Within educational settings, change agents are essential for embedding new content into institutional culture and everyday teaching.

Medical educators are well positioned to act as change agents for the integration of Planetary Health into curricula. Being educators and role models, they influence both formal curricula and students’ understanding of the clinical relevance of sustainability-related topics [17, 18]. Thus, medical educators engagement was identified as a key factor in the successful implementation of PH education [12, 14].

In order to strengthen PH related change agency among medical educators, it is necessary to consider factors beyond their knowledge on a topic. It involves fostering their awareness of the health relevance of climate change, enhancing their self-efficacy, and providing practical pedagogical tools that enable them to integrate PH within the existing curriculum. Trainings addressing these dimensions can empower medical educators to act as multipliers for the longitudinal integration of PH into curricula [15, 18].

The combined demands of clinical duties and research expectations severely limit medical educators’ availability to engage in extensive training, posing a significant barrier to participation [19].

 [20–22] This study aimed to develop and evaluate a compact training for medical educators to enable them to act as change agents for integrating Planetary Health into their curses and thus into the medical curriculum.

## Methods

### Target group, program design, and project context

The training targeted medical educators at Charité – Universitätsmedizin Berlin involved in teaching within the model medical curriculum. Since no institutional training on Planetary Health (PH) had been offered before, participants were assumed to have minimal formal knowledge of PH, aside from informal exposure. Given faculty’s limited time from clinical and research duties, we adopted a concise blended learning format. The training design drew inspiration from the “2slides4future” initiative (https://2slides4future.com/), which encourages academics to add two climate change-related slides to their presentations. Its online templates and examples serve as a useful resource for medical educators integrating Planetary Health content into their teaching.

Guided by Kern’s six-step curriculum development framework [21], we designed a concise training program to help medical educators embed Planetary Health content in their teaching and enhance their role as change agents in the medical curriculum. Following this approach, we introduced Planetary Health content, fostered didactic skills, and allowed time for participants to create their own teaching materials.

Furthermore, we intentionally created space for exchange and networking, with the aim to establish and foster a *community of practice* [23]. Through structured, collective reflection on their roles in the healthcare system, participants were encouraged to build a shared understanding of necessary change, strengthen self-efficacy, and engage in collective learning and support (e.g., shared goals, visible early wins, peer feedback, and ongoing adaptation).

As part of the Climate Adaptation in Transformative Medical Education (CATMED) project, we developed and implemented the training. CATMED, a collaboration between Heidelberg University, Julius-Maximilians-Universität Würzburg, and Charité – Universitätsmedizin Berlin, aims to integrate Planetary Health into medical curricula and promote inter-university collaboration. The Berlin team focused on an intervention targeting medical educators as key stakeholders. The project was funded by the Federal Ministry for the Environment, Nature Conservation, Nuclear Safety and Consumer Protection. All interventions and evaluations conducted within the project were reviewed and approved by the Ethics Committee of Charité – Universitätsmedizin Berlin (approval number: CTO 24–0459).

### Overall and specific learning objectives

Our training aimed to equip medical educators to integrate planetary health into their teaching by combining didactic training in visualization and presentation with foundational Planetary Health knowledge. This objective reflects key recommendations from the AMEE Guide roadmap for faculty development in Planetary Health education [14].

We defined the following overarching learning objectives.

1. Discuss advantages and disadvantages of different visualization formats.
2. Explain requirements for teaching materials in the context of cognitive load theory.
3. Explain the concept of Planetary Health.
4. Reflect on individual and societal health impacts of climate change.
5. Integrate and present Planetary Health topics relevant to their own discipline in an engaging and comprehensible manner.

Reflection on participants’ own attitudes and networking among participants were not explicitly formulated as learning objectives but were considered important implicit goals of the program by the facilitators.

### Teaching methods

To accommodate participants’ limited time, we designed a blended-learning format. Self-directed preparatory and follow-up units for knowledge acquisition and material revision were delivered via an online learning platform. These consisted of one learning unit (LU) before and one LU after the face-to-face session; both units were mandatory to obtain the certificate of completion. The latter included the upload of newly developed teaching material with PH content. The face-to-face session was therefore designed to provide hands-on PH and didactics practice to promote immediate transfer into teaching [24]. Prior research shows such elements enhance practice transfer [25].

The in-person session began with an interactive role-play exercise illustrating climate change’s socially stratified impacts and emotional effects in everyday situations. This was followed by guided reflection on personal vulnerability, didactic input on visualization principles, and Planetary Health content linked directly to participants’ teaching. After the break, participants developed their own teaching materials individually or in pairs. A detailed description of the training can be found in Table 1.

**Table 1:** Detailed description of the training

Phase	Time (min)	Learning objective (participant-centred)	Educational approach	Teaching and learning activities	Key content/ Output
Pre-course Preparation	45	Activate prior knowledge and establish a common baseline understanding of PH	Flipped classroom	Online, on demand, self-study	Introduction to PH focused on interconnectedness of health and environment
Orientation and learning engagement	10	Align expectations, learning goals and course structure	Guided orientation	Visualization on flipchart	Welcome, agenda, learning objectives
Experiential and reflexive engagement	45	Reflect on individual and societal health impacts of climate change through perspective-taking	Experienced based learning, serious game	Role-play “In the middle of society” with guided reflection	Individual health effects of climate change, differential vulnerability and personal impact
Conceptual grounding: design of teaching material	20	Understand requirements for teaching materials in the context of cognitive load theory	Expert input, learning by example	Interactive lecture with worked examples	Slide design principles, cognitive load theory, visual examples
Reflective application and quality criteria	40	Critically analyse teaching materials and derive quality criteria for effective design	Activating reflection	Headstand exercise in small group, plenary discussion	Creation of “worst-practice” slides; development of feedback criteria
	10		Break		
Conceptual grounding: Planetary Health	25	Build a shared conceptual understanding of PH relevant to medical education	Expert input, interactive lecture	Interactive lecture using PowerPoint	Planetary Health framework, planetary boundaries, tipping points, areas of action
Application: identifying entry points in own teaching	25	Identify and critically reflect on opportunities to integrate PH into one’s own courses	Peer teaching, micro-teaching	Tandem work, with on- and offline resources	Identification of starting points and supplementary PH content
	60		Lunch break		
Practical transfer to own teaching	45	Design PH teaching materials for immediate use	Practical learning	Individual or tandem work	Development of 1–2 slides integrating PH content for upcoming course
Micro-teaching and feedback	30	Present PH teaching materials in an engaging and comprehensive manner	Formative assessment	Short presentation, verbal feedback	Presentation of slides,45 + feedback limited to key aspects
Consolidation and evaluation	15	Reflect on learning outcomes and plan transfer into everyday teaching practice	Reflection, formative evaluation	3-3-3 reflection method, plenary discussion	Key takeaways after 3 h, 3 days and 3 months, clarification of open questions
Post-course follow-up	45	Revise and finalize teaching materials for sustainable integration	Flipped classroom, individual work	Submission via online learning platform	Revised slides prepared for use in participants own courses

### Implementation

The program was promoted through multiple channels and faculty networks, with sessions offered at all three university campuses to minimize commuting time. The training was offered within a large university medical institution. Charité reports approximately 5,882 physicians and researchers, although this figure represents only a broad institutional reference point, as not all of these individuals are directly involved in medical teaching or were reached through the recruitment channels used.

### Evaluation

We conducted a descriptive evaluation using Kirkpatrick’s model (Kirkpatrick 1959, 1959a, 1960, 1960a), focusing on content/structure satisfaction (Level 1: reaction), self-reported knowledge gain (Level 2: learning) and transfer to teaching (Level 3: behavior). Pre-training assessments covered PH motivation, prior knowledge, and demographics (age, gender, teaching role, experience). Information on medical disciplines was collected to characterize the participant group but are not reported in detail to protect participant anonymity, given the small sample size and the risk of re-identification. A Level 4 evaluation of student’s outcomes (results) was neither feasible nor methodologically sound, given the heterogeneous nature of change across faculty courses and cohorts. Post-workshop evaluation used self-developed 5-point Likert items (prior knowledge, motivation, satisfaction, knowledge gain, intended transfer), internally validated through learning objectives and two open-ended feedback questions.

Transfer was assessed via documented PH teaching materials and a 12-month follow-up survey; quantitative data analyzed descriptively in Microsoft Excel (2024). The evaluation was conducted online using Microsoft Forms. Participants provided written informed consent prior to participation in the evaluation and data analysis. The questionnaire is provided in *Appendix 1*.

## Results

Three trainings were conducted in February, August, and September 2024 (6, 8, and 4 participants, respectively). A planned October workshop at the smallest campus was cancelled due to low enrollment (two registrations).

### Implementation experiences

The training was delivered via interdisciplinary team-teaching (2–3 facilitators, including medical/geography PH experts). The first implementation showed that strict time management—especially precise input timing—was essential for smooth transitions to application phases, which needed adequate time for contextual adaptation. Despite access to examples and materials, many participants struggled to develop 1–2 teaching slides during self-study. The presence of two facilitators in small groups provided individual guidance, actively utilized by participants and essential for workshop success.

### Participants

A total of 18 medical educators participated in three workshops. Seventeen (94%) completed the immediate post-workshop survey (10 females, 7 males, ages 28–57 years). Teaching experience varied: 7 had ≤ 3 years, 9 had > 10 years of teaching experience. Participants represented diverse medical faculty disciplines with some educators responsible for up to 20 teaching sessions per semester. Participants also differed in their teaching roles and degree of influence on teaching design. Five participants held roles with greater decision-making scope, for example as teaching coordinators or in relevant institutional teaching roles. However, participants generally had limited direct influence on formal curriculum development or curricular learning objectives. Their scope of action primarily concerned the selection of teaching content and methods within existing teaching formats, as well as providing feedback to curriculum planning processes. All reported strong Planetary Health interest (agree: *n* = 5; strongly agree: *n* = 12) and climate role model self-perception (agree: *n* = 10; strongly agree: *n* = 6), with 8/17 (47%) already attempting to integrate PH content into their teaching beforehand; 9 (50%) completed the 12-month follow-up.

### Satisfaction (Kirkpatrick level 1: reaction)

**Fig. 1:**
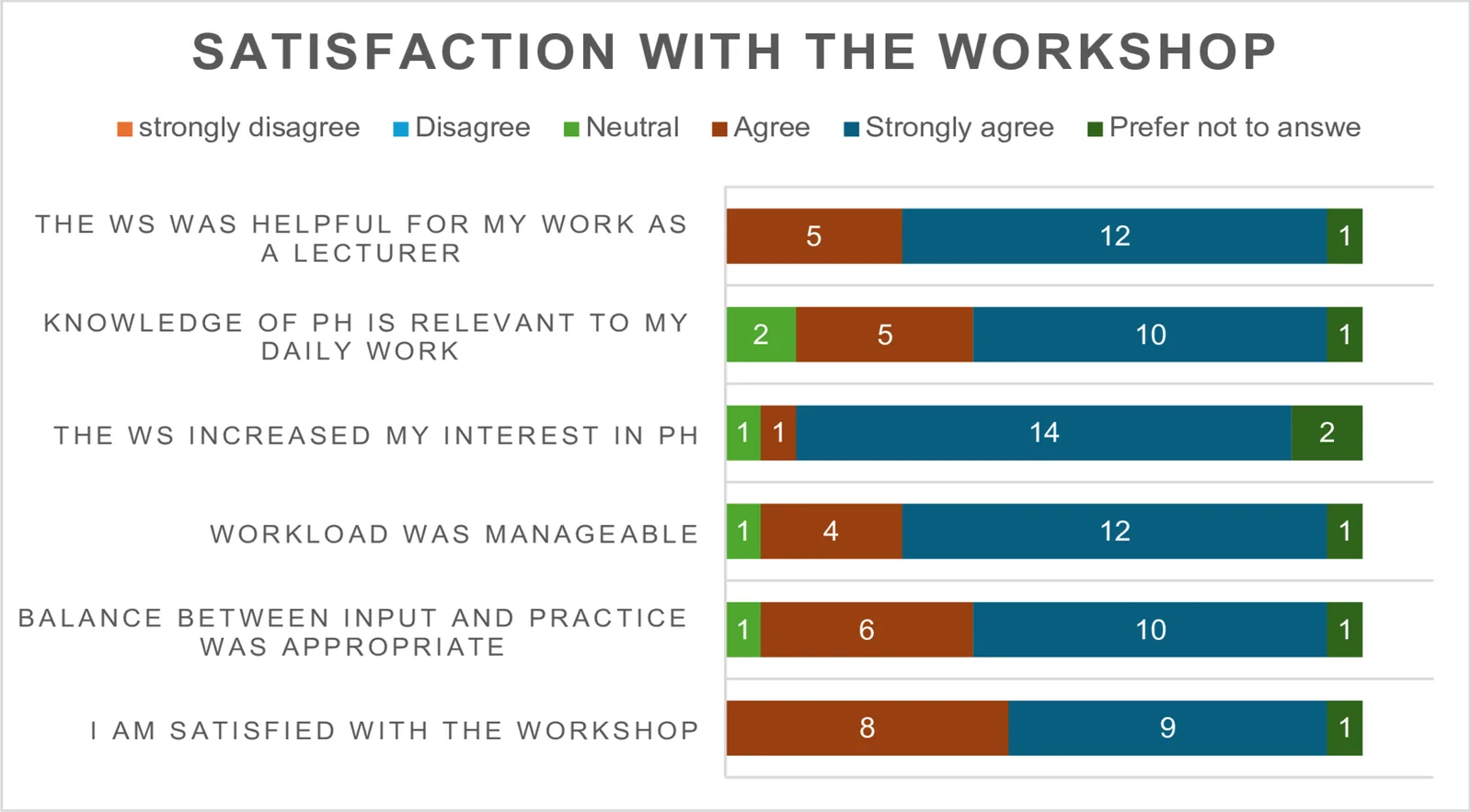
Satisfaction with the workshop

Overall, participants reported high satisfaction with the workshop and with the balance between instructional input and practical exercises. The workshop increased interest in Planetary Health and was perceived as relevant to participants’ teaching activities (see Fig. 1).

### Subjective learning outcomes (Kirkpatrick level 2: learning)

Participants rated Planetary Health knowledge gain as moderate (*n* = 9) to high (*n* = 8). Knowledge gains relevant to their teaching were moderate (*n* = 7) or high/very high (*n* = 9). Post-training, 9 rated their integration ability as high/very high, 4 as moderate. Fifteen out of seventeen participants found the PH-didactics combination helpful for teaching integration.

### Self-reported transfer into teaching practice (Kirkpatrick level 3: behaviour)

Immediately after the workshop, most participants completed the follow-up phase and reported intentions to integrate the developed teaching materials and additional Planetary Health content into their teaching, as well as to share the materials with colleagues.

**Fig. 2:**
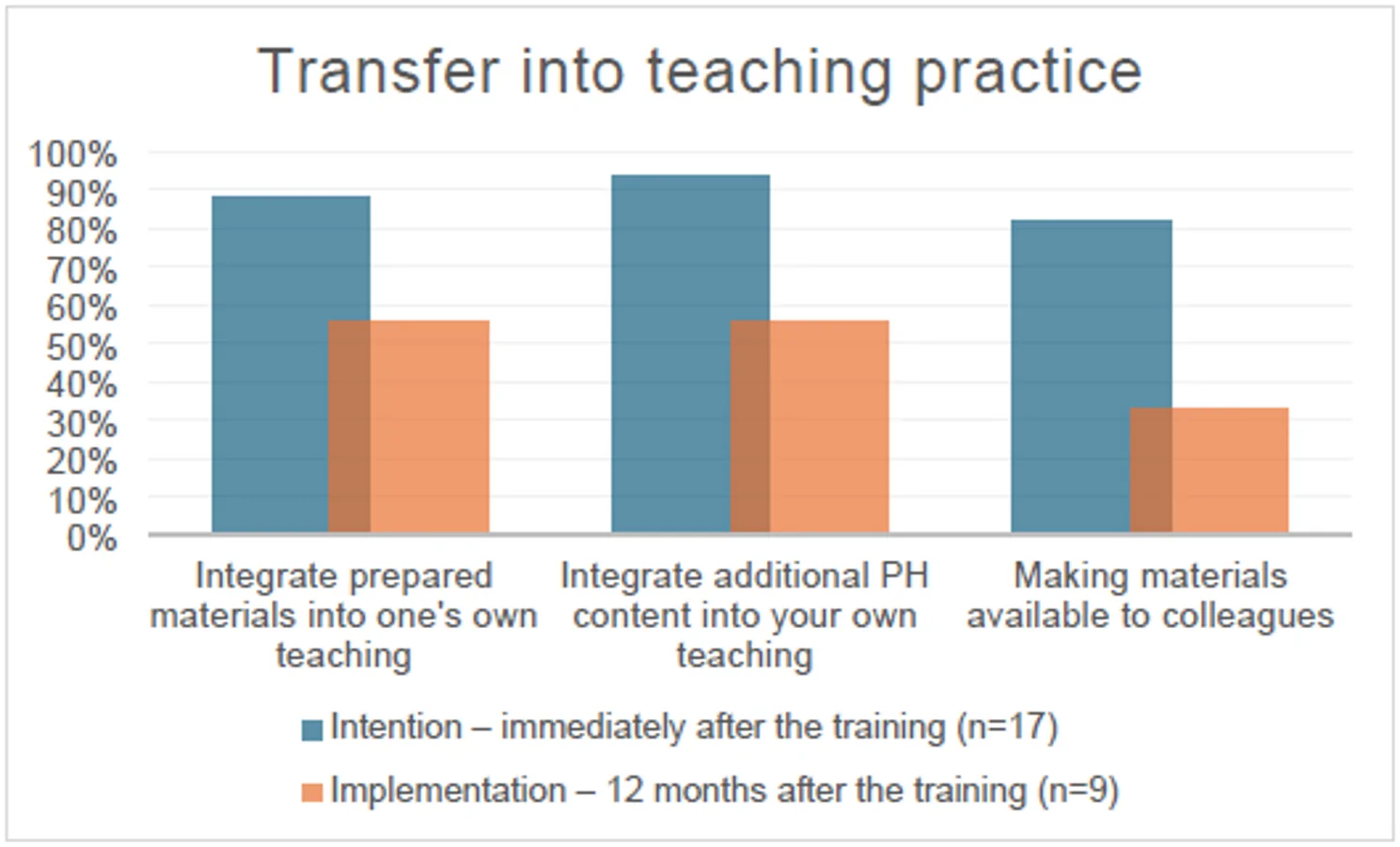
Transfer into teaching practice

At the 12-month follow-up, 9/18 participants responded; 5 had integrated materials into their teaching and 3 had shared them with colleagues (Fig. 2).

### Perceived contextual factors influencing transfer

Perceived negative reactions from the surrounding environment appeared to play only a minor role in the transfer process. Only a small number of participants expected resistance from colleagues or students when integrating Planetary Health content into their teaching (*n* = 3 and *n* = 2, respectively). Eight of the nine respondents who participated in the 12-month follow-up reported having experienced predominantly positive reactions from colleagues and students.

Perceived negative environmental reactions played only a minor role for transfer. Few participants expected resistance from colleagues (*n* = 3) or students (*n* = 2) when integrating Planetary Health into their teaching. Among 12-month follow-up respondents (*n* = 9), 8 reported predominantly positive reactions from both groups.

## Discussion

### Project objectives

Medical educators with diverse teaching experience and disciplines—all with pre-existing Planetary Health interest—attended the training. They reported high satisfaction with its structure, content, and everyday teaching relevance, rating overall PH knowledge gain as moderate to high and acquired integration competence as high.

All but one lecturer completed follow-up and developed course-specific teaching materials. Most intended to integrate Planetary Health content and share with colleagues. At 12-month follow-up (*n* = 9/18), half had implemented PH content, though few shared materials; negative reactions played a minimal role. Low participation typical for Charité non-mandatory trainings. The one-day format didn’t increase participation numbers, but small groups enabled intensive transfer support, enabling team teaching and more resources for all sessions. Based on first-training experiences, team teaching (originally planned solely for that session) proved necessary and was implemented across all workshops, increasing personnel and resource requirements.

### Comparison with other educational projects

Despite the short duration of the workshop, nearly all participants were able to develop teaching materials suitable for use in their own courses.

Consistent with prior research, learning transfer to practice proved challenging, depending on competence, autonomy, perceived value, and supportive context [24]. While most medical educators intended to implement the materials, only half had done so at follow-up. This discrepancy can be interpreted in light of the intention-behavior gap, which describes the well-known difference between intended and enacted behavior. In teaching contexts, such gaps may occur because translating pedagogical intentions into classroom practice depends not only on knowledge and motivation, but also on available teaching opportunities, curricular fit, institutional structures, and teachers’ scope of action [25]. As Kirkpatrick Level 3 outcomes (actual behavior change) are rarely assessed, it’s unclear if other formats achieve higher implementation rates.

The related “knowledge-to-action gap”—encompassing motivational, systemic, emotional, and social dimensions [24]—may further explain why intentions do not always translate into implementation. According to the evaluation, participants felt sufficiently competent to implement PH into their teaching. To sustain implementation motivation, explicit action plans prove beneficial [26], which informed the workshop’s didactic concept for transfer preparation. As fear of negative reactions hinders behavior change [24], a community of practice approach [27] was implemented to enhance social acceptability. Thus, the workshop promoted lecturer networking for collective rather than isolated action, with few negative reactions reported at follow-up.

Potential factors that may have hindered implementation for some participants include systemic constraints, such as highly regulated curricula that offer limited encouragement for individual initiative, as well as a lack of institutional incentives or recognition for curricular innovation. Although lecturers could adapt teaching content and methods within their own existing teaching formats, they generally had limited direct influence on formal curriculum development or curricular learning objectives. This may have constrained the translation of newly developed PH materials into sustained curricular integration, despite high motivation and perceived relevance. A comparable integrative approach has previously been described by Flaegel et al. [20] in an elective course for medical students who are interested in teaching. It encompassed a whole semester and supported students to develop an online lesson with planetary health content. The impact on medical school curricula or teaching practice was not evaluated.

### Outlook and future development

Although personnel-intensive, the time commitment required from both facilitators and participants was limited. Faculty development programs aiming to change teaching behavior are often associated with substantially higher resource demands [28]. The community-of-practice approach has been discussed as a key success factor for sustainable faculty development. Networking among medical educators was therefore a central component of the program and a primary reason for implementing the training as an in-person format. To support networking beyond the individual workshops, a Planetary Health teaching working group was established within the project and successfully embedded under the umbrella of the Charité Climate Network.

In the second quarter of 2025, an increasing number of medical educators requested training opportunities related to PH, underscoring both the need and interest for structural integration of such formats. However, following the end of project funding, additional workshops could not be offered. A long-term e-learning format was considered but abandoned due to the critical importance of personal interaction. Key success factors included peer exchange, individualized material development support, group feedback on teaching materials, and lecturer networking.

### Transferability and broader implications

Beyond the local context, this study demonstrates that a compact, transfer-oriented training can successfully support medical educators in integrating PH content into their teaching. While the intervention was implemented within a single institution, its core design principles—low-threshold participation, explicit preparation for transfer, and a focus on change agency within a community-of-practice—are not institution-specific.

Our findings suggest that similar initiatives should not rely on faculty motivation alone. Although the training enabled participants to develop course-specific PH teaching materials, subsequent implementation probably depended on available teaching opportunities, curricular fit, and educators’ degree of influence within existing teaching formats. Future faculty development programs should therefore combine material development during the training with explicit implementation support, such as structured follow-up sessions, peer feedback, protected time for teaching innovation, and coordination with curriculum committees or module leads. Embedding such initiatives within institutional structures may help reduce the gap between individual motivation and sustained curricular integration.

Medical faculties that already offer training for faculty members may readily adapt this approach to their local contexts, integrating PH content into their existing training programs. Given the widespread time constraints and competing clinical/research demands across medical schools nationally and internationally, this model appears transferable to diverse educational contexts.

### Limitations

This study has several limitations. First, the small sample size and voluntary participation likely introduced selection bias toward medical educators with pre-existing Planetary Health interest. Second, most outcomes relied on self-reported measures, which are susceptible to social desirability bias and may not reflect objective teaching behavior changes. Although, the immediate post-workshop items captured perceived transfer potential and intentions to integrate and share PH-related teaching materials, the evaluation design does not allow causal conclusions about intervention-related change. Third, the moderate 12-month follow-up response rate (50%) limits conclusions about long-term implementation. Because surveys were not linked to preserve participant anonymity, we were unable to compare demographic characteristics between immediate post-workshop and 12-month follow-up respondents or assess whether specific participant characteristics were associated with follow-up participation. In addition, barriers to implementation were not systematically assessed at follow-up; therefore, potential reasons for non-implementation, such as time constraints, curricular fit, limited teaching opportunities, or restricted decision-making authority, can only be inferred from the broader implementation context. Finally, the evaluation did not include qualitative interviews or a more detailed survey to explore barriers to and facilitators of PH implementation in depth. Such data would be valuable to strengthen the transferability of findings and inform future faculty development initiatives. A qualitative follow-up study with lecturers is currently underway to address these questions.

However, a strength is our assessment of Kirkpatrick Level 3 outcomes (actual behavior change)—rarely evaluated in faculty development studies—which provides valuable insights into real-world transfer despite these limitations. Kirkpatrick Level 4 (student results/clinical outcomes) was not feasible due to heterogeneous curriculum-wide implementation. Future research could target Level 4 once Planetary Health content achieves more consistent curricular embedding.

## Conclusion

This study demonstrates that medical educators are key stakeholders and potential change agents for the integration of Planetary Health content into medical curricula. Institutional faculty trainings customized to medical educators’ concrete needs and limited time resources, with practical implementation support, could successfully facilitate Planetary Health curricular integration. To ensure sustainable impact beyond individual training sessions, ongoing networking among medical educators—such as through communities of practice—appears to be a promising strategy.

## Supplementary Information

Supplementary Material 1.

## Data Availability

The datasets generated and analyzed during the current study are not publicly available due to data protection and privacy considerations but are available from the corresponding author on reasonable request.

## References

[1] WHO. WHO factsheet on climate change and health. 2023. Cited 2025 Aug 1. Climate change. Available from: https://www.who.int/news-room/fact-sheets/detail/climate-change-and-health.

[2] Romanello M, Di Napoli C, Drummond P, Green C, Kennard H, Lampard P, et al. The 2022 report of the Lancet Countdown on health and climate change: health at the mercy of fossil fuels. Lancet. 2022;400(10363):1619–54. 10.1016/S0140-6736(22)01540-9 . PubMed PMID: 36306815; PubMed Central PMCID: PMC7616806. 36306815 10.1016/S0140-6736(22)01540-9 PMC7616806

[3] Prescott SL, Logan AC, Albrecht G, Campbell DE, Crane J, Cunsolo A, et al. The Canmore Declaration: Statement of Principles for Planetary Health. Challenges. 2018;9(2):2. 10.3390/challe9020031.

[4] Mlinarić M, Moebus S, Betsch C, Hertig E, Schröder J, Loss J, et al. Climate change and public health in Germany - A synthesis of options for action from the German status report on climate change and health 2023. J Health Monit. 2023;8(Suppl 6):57–85. /11774 PubMed PMID: 38105793; PubMed Central PMCID: PMC10722518. 10.25646/11774 PMC10722518 38105793

[5] Chase H, Hampshire K, Tun S. Improving the medical curriculum on planetary health and sustainable healthcare. 2022. 10.1136/bmj.o209. 10.1136/bmj.o209 35078768

[6] Visser EH, Oosterveld B, Slootweg IA, Vos HMM, Adriaanse MA, Schoones JW, et al. The Development and Characteristics of Planetary Health in Medical Education: A Scoping Review. Acad Med. 2024;99(10):1155. 10.1097/ACM.0000000000005796. 38954502 10.1097/ACM.0000000000005796

[7] Wabnitz K, Schwienhorst-Stich EM, Asbeck F, Fellmann CS, Gepp S, Leberl J, et al. National Planetary Health learning objectives for Germany: A steppingstone for medical education to promote transformative change. Front Public Health. 2023;10:1093720. 10.3389/fpubh.2022.1093720 . PubMed PMID: 36937826; PubMed Central PMCID: PMC10015604. 36937826 10.3389/fpubh.2022.1093720 PMC10015604

[8] Schwienhorst-Stich EM, Kropff D, Kersken K, König S, Leutritz T, Parisi S, et al. Das Wahlpflichtfach Planetare Gesundheit: Klima.Umwelt.Gesundheit an der Medizinischen Fakultät Würzburg – Konzeption, didaktische Methoden und Evaluationsergebnisse. Zeitschrift für Evidenz Fortbildung und Qualität im Gesundheitswesen. 2024;186:92–103. 10.1016/j.zefq.2023.12.001. 38575437 10.1016/j.zefq.2023.12.001

[9] Lemke D, Holtz S, Gerber M, Amberger O, Schütze D, Müller B, et al. From niche topic to inclusion in the curriculum – design and evaluation of the elective course climate change and health. GMS J Med Educ. 2023;40(3):Doc31. 10.3205/zma001613 . PubMed PMID: 37377570; PubMed Central PMCID: PMC10291346. 37377570 10.3205/zma001613 PMC10291346

[10] Grieco F, Parisi S, Simmenroth A, Eichinger M, Zirkel J, König S, et al. Planetary health education in undergraduate medical education in Germany: results from structured interviews and an online survey within the national PlanetMedEd Project. Front Med. 2025;11:1507515. 10.3389/fmed.2024.1507515. 10.3389/fmed.2024.1507515 PMC11851124 40007820

[11] Phillips-Wilson T, Khadke S, Mares A, Lawrence J, Shah J, Rajagopalan S, et al. Integrating Planetary Health Into Medical Education: An ACC Medical Student Leadership Perspective. JACC: Adv. 2024;3(12, Part 1):101366. 10.1016/j.jacadv.2024.101366. 39583868 10.1016/j.jacadv.2024.101366 PMC11585745

[12] Selvam R, Séguin N, Zhang L, Lacaille-Ranger A, Sikora L, Raiche I, et al. International Planetary Health Education in Undergraduate and Graduate Medical Curricula: A Scoping Review. J Grad Med Educ. 2024;16(6 Suppl):58–68. 10.4300/JGME-D-24-00027.1 . PubMed PMID: 39677906; PubMed Central PMCID: PMC11644581. 39677906 10.4300/JGME-D-24-00027.1 PMC11644581

[13] Simon J, Parisi S, Wabnitz K, Simmenroth A, Schwienhorst-Stich EM. Ten characteristics of high-quality planetary health education-Results from a qualitative study with educators, students as educators and study deans at medical schools in Germany. Front Public Health. 2023;11:1143751. 10.3389/fpubh.2023.1143751 . PubMed PMID: 37181714; PubMed Central PMCID: PMC10166869. 37181714 10.3389/fpubh.2023.1143751 PMC10166869

[14] Shaw E, Walpole S, McLean M, Alvarez-Nieto C, Barna S, Bazin K, et al. AMEE Consensus Statement: Planetary health and education for sustainable healthcare. Med Teach. 2021;43(3):272–86. 2020.1860207 PubMed PMID: 33602043. 33602043 10.1080/0142159X.2020.1860207

[15] Fullan M, editor. The NEW meaning of educational change. New York, NY: Teachers College; 2016. p. 1.

[16] Biesta G, Priestley M, Robinson S. The role of beliefs in teacher agency. Teachers Teach. 2015;21(6):624–40. 10.1080/13540602.2015.1044325.

[17] Frenk J, Chen L, Bhutta ZA, Cohen J, Crisp N, Evans T, et al. Health professionals for a new century: transforming education to strengthen health systems in an interdependent world. Lancet. 2010;376(9756):1923–58. 10.1016/S0140-6736(10)61854-5. 21112623 10.1016/S0140-6736(10)61854-5

[18] Steinert Y, Mann K, Anderson B, Barnett BM, Centeno A, Naismith L, et al. A systematic review of faculty development initiatives designed to enhance teaching effectiveness: A 10-year update: BEME Guide 40. Med Teach. 2016;38(8):769–86. 10.1080/0142159X.2016.1181851. 27420193 10.1080/0142159X.2016.1181851

[19] Sachdeva AK. Faculty Development and Support Needed to Integrate the Learning of Prevention in the Curricula of Medical Schools. Acad Med. 2000;75(Supplement):S35–42. 10.1097/00001888-200007001-00006. 10.1097/00001888-200007001-00006 10926039

[20] Flägel K, Manke M, Zimmermann K, Wagener S, Pante SV, Lehmann M, et al. Planetary health as a main topic for the qualification in digital teaching - a project report. GMS J Med Educ. 2023;40(3):Doc35. 10.3205/zma001617 . PubMed PMID: 37377576; PubMed Central PMCID: PMC10291345. 37377576 10.3205/zma001617 PMC10291345

[21] Thomas PA, Kern DE, Hughes MT, Tackett SA, Chen BY, editors. Curriculum development for medical education: a six-step approach. Fourth edition. Baltimore: Johns Hopkins University Press. 2022;1. https://lccn.loc.gov/2021048343.

[22] Thomas P. Curriculum Development for Medical Education [Internet]. Johns Hopkins University Press. 2016. Cited 2026 Jan 20. Available from: https://muse.jhu.edu/book/4460010.1353/book.44600.

[23] Wenger-Trayner B. a brief overview of the concept and its uses. Communities of practice. 2015.

[24] Kahlke RM, McConnell MM, Wisener KM, Eva KW. The disconnect between knowing and doing in health professions education and practice. Adv Health Sci Educ Theory Pract. 2020;25(1):227–40. 10.1007/s10459-019-09886-5 . PubMed PMID: 30904958. 30904958 10.1007/s10459-019-09886-5

[25] Dittrich AK, Eloff I. The intention-behaviour gap: Three case studies of the application of general pedagogical knowledge. 2022;121–36. 10.17159/8a4z9c80.

[26] Boekaerts M, Maes S, Karoly P. Self-Regulation Across Domains of Applied Psychology: Is there an Emerging Consensus? Applied Psychology. Int Rev. 2005;54(2):149–54. 10.1111/j.1464-0597.2005.00201.x.

[27] Lave J, Wenger E, Situated Learning. Legitimate Peripheral Participation. 1st edn. Cambridge University Press. 1991. Cited 2026 Jan 20. Available from: https://www.cambridge.org/core/product/identifier/9780511815355/type/book10.1017/CBO9780511815355.

[28] Haas M, Triemstra J, Tam M, Neuendorf K, Reckelhoff K, Gottlieb-Smith R, et al. A decade of faculty development for health professions educators: lessons learned from the Macy Faculty Scholars Program. BMC Med Educ. 2023;23(1):185. 10.1186/s12909-023-04155-x. 36973722 10.1186/s12909-023-04155-x PMC10041479

