# Supplementary material for: Empowering medical educators to teach planetary health: development and outcomes of a curriculum integration training
Source: BMC Med Educ. 2026 Aug 18;26:1339. doi: 10.1186/s12909-026-10170-5 (PMC13487975; doi:10.1186/s12909-026-10170-5)
Supplement: Supplementary file 1 [file 12909_2026_10170_MOESM1_ESM.docx]

**Questionnaire for Participants of the Didactics Training directly after training**

This questionnaire is intended to help assess and improve the quality of teaching. We therefore kindly ask you to complete it carefully. Your anonymity is guaranteed during both the survey and the evaluation.

Training period: ____________________________

Date of survey: ____________________________

**Demographics**

1. Age: ____________________________

2. Gender: ☐ Male	☐ Female	☐ Diverse

3. Which subjects do you teach?

Please specify: ____________________________

4. How many years have you been teaching?

Please specify: ____________________________

5. Approximately how many courses do you teach per semester?

☐ 1–5	☐ 6–10	☐ 11–15	☐ 16–20	☐ >20

Please indicate for each statement below the extent to which you personally agree. You may choose between five response options ranging from “strongly agree” to “strongly disagree.” Please clearly mark one option for each statement. Please answer all questions yourself.

**Motivation prior to the training**

6. I attended this training because I am interested in didactics.

☐ Strongly agree	☐ Rather agree	☐ Partly agree	☐ Rather disagree	☐ Strongly disagree	☐ No answer

7. I attended this training because I am interested in Planetary Health.

☐ Strongly agree	☐ Rather agree	☐ Partly agree	☐ Rather disagree	☐ Strongly disagree	☐ No answer

8. I have previously participated in further training on Planetary Health.

☐ Strongly agree	☐ Rather agree	☐ Partly agree	☐ Rather disagree	☐ Strongly disagree	☐ No answer

9. I already try to integrate Planetary Health into my teaching.

☐ Strongly agree	☐ Rather agree	☐ Partly agree	☐ Rather disagree	☐ Strongly disagree	☐ No answer

10. As a lecturer, I serve as a role model regarding climate change.

☐ Strongly agree	☐ Rather agree	☐ Partly agree	☐ Rather disagree	☐ Strongly disagree	☐ No answer

**Satisfaction**

11. The content was appropriately illustrated (e.g., through examples, visualizations, etc.).

☐ Strongly agree	☐ Rather agree	☐ Partly agree	☐ Rather disagree	☐ Strongly disagree	☐ No answer

12. The balance between theoretical input and practical exercises was appropriate.

☐ Strongly agree	☐ Rather agree	☐ Partly agree	☐ Rather disagree	☐ Strongly disagree	☐ No answer

13. The workload was appropriate for the scope of the training.

☐ Strongly agree	☐ Rather agree	☐ Partly agree	☐ Rather disagree	☐ Strongly disagree	☐ No answer

14. The course increased my interest in Planetary Health.

☐ Strongly agree	☐ Rather agree	☐ Partly agree	☐ Rather disagree	☐ Strongly disagree	☐ No answer

15. I find it relevant for my daily work to acquire in-depth knowledge about Planetary Health.

☐ Strongly agree	☐ Rather agree	☐ Partly agree	☐ Rather disagree	☐ Strongly disagree	☐ No answer

16. I would like more advanced training opportunities in didactics.

☐ Strongly agree	☐ Rather agree	☐ Partly agree	☐ Rather disagree	☐ Strongly disagree	☐ No answer

17. I would like more advanced training opportunities in Planetary Health.

☐ Strongly agree	☐ Rather agree	☐ Partly agree	☐ Rather disagree	☐ Strongly disagree	☐ No answer

18. The content of the training is relevant to my professional daily work.

☐ Strongly agree	☐ Rather agree	☐ Partly agree	☐ Rather disagree	☐ Strongly disagree	☐ No answer

19. I see the training as an opportunity to improve my teaching.

☐ Strongly agree	☐ Rather agree	☐ Partly agree	☐ Rather disagree	☐ Strongly disagree	☐ No answer

20. The course was helpful for my work as a lecturer.

☐ Strongly agree	☐ Rather agree	☐ Partly agree	☐ Rather disagree	☐ Strongly disagree	☐ No answer

21. I believe that participating in the training was worthwhile for me personally.

☐ Strongly agree	☐ Rather agree	☐ Partly agree	☐ Rather disagree	☐ Strongly disagree	☐ No answer

**Satisfaction and Use of ePlanet**

22. This game changed my thinking about the social and environmental causes of diseases.

☐ Strongly agree	☐ Rather agree	☐ Partly agree	☐ Rather disagree	☐ Strongly disagree	☐ No answer

23. I consider this game a useful tool for introducing the topic of Planetary Health.

☐ Strongly agree	☐ Rather agree	☐ Partly agree	☐ Rather disagree	☐ Strongly disagree	☐ No answer

24. By playing ePlanet, I learned something new.

☐ Strongly agree	☐ Rather agree	☐ Partly agree	☐ Rather disagree	☐ Strongly disagree	☐ No answer

25. After playing the game, I feel more motivated to talk about Planetary Health topics with colleagues, patients, and students.

☐ Strongly agree	☐ Rather agree	☐ Partly agree	☐ Rather disagree	☐ Strongly disagree	☐ No answer

26. I intend to integrate ePlanet into at least one of my courses.

☐ Strongly agree	☐ Rather agree	☐ Partly agree	☐ Rather disagree	☐ Strongly disagree	☐ No answer

**Please assess your current level of knowledge on various topics related to climate and environment. Note: The scale changes here.**

27. I would rate my knowledge gain regarding Planetary Health as follows:

☐ Very high	☐ High	☐ Moderate	☐ A little	☐ Unchanged	☐ No answer

28. I would rate my knowledge gain regarding the interaction between human and planetary health as follows:

☐ Very high	☐ High	☐ Moderate	☐ A little	☐ Unchanged	☐ No answer

29. I would rate my knowledge gain regarding Planetary Health in areas relevant to my teaching as follows:

☐ Very high	☐ High	☐ Moderate	☐ A little	☐ Unchanged	☐ No answer

30. After the training, I would rate my ability to integrate Planetary Health content into my teaching as follows:

☐ Very high	☐ High	☐ Moderate	☐ A little	☐ Unchanged	☐ No answer

**How will the training influence your teaching in the future?**

31. The relevance of the topics covered became clear to me (especially the relevance of integrating Planetary Health into my teaching).

☐ Strongly agree	☐ Rather agree	☐ Partly agree	☐ Rather disagree	☐ Strongly disagree	☐ No answer

32. By linking Planetary Health with didactic methods, I can better integrate what I have learned into my teaching.

☐ Strongly agree	☐ Rather agree	☐ Partly agree	☐ Rather disagree	☐ Strongly disagree	☐ No answer

33. I intend to integrate the slides I created into at least one of my courses.

☐ Strongly agree	☐ Rather agree	☐ Partly agree	☐ Rather disagree	☐ Strongly disagree	☐ No answer

34. I plan to develop additional Planetary Health content and integrate it into at least one of my courses.

☐ Strongly agree	☐ Rather agree	☐ Partly agree	☐ Rather disagree	☐ Strongly disagree	☐ No answer

35. Other colleagues involved in teaching may use the slides I created with Planetary Health content for their teaching.

☐ Strongly agree	☐ Rather agree	☐ Partly agree	☐ Rather disagree	☐ Strongly disagree	☐ No answer

36. I would like to share the knowledge I gained with other colleagues involved in teaching at my institute.

☐ Strongly agree	☐ Rather agree	☐ Partly agree	☐ Rather disagree	☐ Strongly disagree	☐ No answer

37. I expect to encounter resistance from colleagues when trying to integrate Planetary Health content into my teaching.

☐ Strongly agree	☐ Rather agree	☐ Partly agree	☐ Rather disagree	☐ Strongly disagree	☐ No answer

38. I expect to encounter resistance from students when integrating Planetary Health content into my teaching.

☐ Strongly agree	☐ Rather agree	☐ Partly agree	☐ Rather disagree	☐ Strongly disagree	☐ No answer

**Comments / Suggestions for Improvement:**

What I particularly liked about the training:

___________________________________________________________________________________________

___________________________________________________________________________________________

___________________________________________________________________________________________

What I would have preferred to be different:

___________________________________________________________________________________________

___________________________________________________________________________________________

___________________________________________________________________________________________

**Thank you for taking the time to participate in this survey.**

Your participation is of great importance to us, and we would greatly appreciate the opportunity to contact you for a follow-up survey.

**Background:**

So far, you have shared your insights from the training and thereby contributed to answering our research question:

“Integration of Planetary Health aspects into medical education. A mixed-methods study on attitudes and needs of lecturers.”

We would be pleased to include you in our follow-up survey in order to examine insights, changes, and developments in teaching.

**Questionnaire follow-up survey**

Dear Lecturer,

Thank you very much for deciding to participate in our follow-up survey on the topic:

“Integration of Planetary Health Aspects into Medical Education. A Mixed-Methods Study on Attitudes and Needs of Lecturers.”

The following questions are intended to provide an overview of the extent to which your participation in the lecturer training

“Creating Learning-Effective Teaching Materials – Didactic Tips and Practical Implementation Using the Example of Planetary Health for My Teaching”

has influenced your teaching. This survey is explicitly not intended to evaluate your teaching performance, but rather to help us understand how we can better support you in implementing teaching with and about Planetary Health. We therefore kindly ask you to complete this questionnaire carefully. Your anonymity is guaranteed during both the survey and its evaluation.

Date of survey: ______________________________

1. I have integrated the slides I created during the training into at least one of my courses.

☐ Strongly agree	☐ Rather agree	☐ Partly agree	☐ Rather disagree	☐ Strongly disagree	☐ No answer

2. I have developed additional content on Planetary Health and integrated it into at least one of my courses.

☐ Strongly agree	☐ Rather agree	☐ Partly agree	☐ Rather disagree	☐ Strongly disagree	☐ No answer

3. Through the training, I was able to integrate Planetary Health content into my teaching in a didactically accessible way.

☐ Strongly agree	☐ Rather agree	☐ Partly agree	☐ Rather disagree	☐ Strongly disagree	☐ No answer

4. Other colleagues involved in teaching were allowed to use the Planetary Health-related slides I created for their own teaching.

☐ Strongly agree	☐ Rather agree	☐ Partly agree	☐ Rather disagree	☐ Strongly disagree	☐ No answer

5. I have passed on the knowledge I acquired to other colleagues involved in teaching at my institute.

☐ Strongly agree	☐ Rather agree	☐ Partly agree	☐ Rather disagree	☐ Strongly disagree	☐ No answer

6. I experienced resistance from colleagues when I attempted to integrate Planetary Health content into my teaching.

☐ Strongly agree	☐ Rather agree	☐ Partly agree	☐ Rather disagree	☐ Strongly disagree	☐ No answer

7. I experienced resistance from students when I integrated Planetary Health content into my teaching.

☐ Strongly agree	☐ Rather agree	☐ Partly agree	☐ Rather disagree	☐ Strongly disagree	☐ No answer

8. I received positive feedback from colleagues when I attempted to integrate Planetary Health content into my teaching.

☐ Strongly agree	☐ Rather agree	☐ Partly agree	☐ Rather disagree	☐ Strongly disagree	☐ No answer

9. I received positive feedback from students when I integrated Planetary Health content into my teaching.

☐ Strongly agree	☐ Rather agree	☐ Partly agree	☐ Rather disagree	☐ Strongly disagree	☐ No answer

10. Other colleagues use the teaching materials on Planetary Health that I created.

☐ Strongly agree	☐ Rather agree	☐ Partly agree	☐ Rather disagree	☐ Strongly disagree	☐ No answer

11. Outside of my courses, I have also attempted to address aspects of Planetary Health in discussions with students.

☐ Strongly agree	☐ Rather agree	☐ Partly agree	☐ Rather disagree	☐ Strongly disagree	☐ No answer

12. Outside of my courses, I have also attempted to address aspects of Planetary Health in discussions with colleagues.

☐ Strongly agree	☐ Rather agree	☐ Partly agree	☐ Rather disagree	☐ Strongly disagree	☐ No answer

13. I would have liked more content on Planetary Health in order to sustainably enrich my teaching.

☐ Strongly agree	☐ Rather agree	☐ Partly agree	☐ Rather disagree	☐ Strongly disagree	☐ No answer
